# Mortality and causes of death in a national sample of type 2 diabetic patients in Korea from 2002 to 2013

**DOI:** 10.1186/s12933-016-0451-0

**Published:** 2016-09-13

**Authors:** Yu Mi Kang, Ye-Jee Kim, Joong-Yeol Park, Woo Je Lee, Chang Hee Jung

**Affiliations:** 1 Department of Internal Medicine, Asan Medical Center, University of Ulsan College of Medicine, 88, Olympic-ro 43-gil, Songpa-gu, Seoul, South Korea; 2 Department of Clinical Epidemiology and Biostatistics, Asan Medical Center, University of Ulsan College of Medicine, 88, Olympic-ro 43-gil, Songpa-gu, Seoul, South Korea

**Keywords:** Type 2 diabetes, Mortality, Mortality rate, Standardized mortality rate

## Abstract

**Background:**

We aimed to investigate the mortality rate (MR), causes of death and standardized mortality ratio (SMR) in Korean type 2 diabetic patients from 2002 to 2013 using data from the Korean National Health Insurance Service-National Sample Cohort (NHIS-NSC).

**Methods:**

From this NHIS-NSC, we identified 29,807 type 2 diabetic subjects from 2002 to 2004. Type 2 diabetes was defined as a current medication history of anti-diabetic drugs and the presence of International Classification of Diseases (ICD)-10 codes (E11–E14) as diagnosis. Specific causes of death were recorded according to ICD-10 codes as the following: diabetes, malignant neoplasm, disease of the circulatory system, and other causes.

**Results:**

A total of 7103 (23.8 %) deaths were recorded. The MR tended to increase with age. In particular, the ratio of MR for men versus women was the highest in their 40s–50s. The overall SMR was 2.32 and the SMRs attenuated with increasing age. The causes of death ascribed to diabetes, malignant neoplasm, ischemic heart disease, cerebrovascular disease, and other causes were 22.0, 24.8, 6.2, 11.2 and 31.3 %, respectively. The SMRs according to each cause of death were 9.73, 1.76, 2.60, 2.04 and 1.89, respectively.

**Conclusions:**

The MRs among type 2 diabetic subjects increased with age, and diabetic men exhibited a higher mortality risk than diabetic women in Korea. Subjects with type 2 diabetes exhibited an excess mortality when compared with the general population. Approximately 78.0 % of the diabetes-related deaths was not ascribed to diabetes, and malignant neoplasm was the most common cause of death among those not recorded as diabetes.

**Electronic supplementary material:**

The online version of this article (doi:10.1186/s12933-016-0451-0) contains supplementary material, which is available to authorized users.

## Background

Individuals with diabetes have higher all-cause mortality rates (MRs) compared to similar people without diabetes [1], primarily due to the development of cardiovascular disease (CVD) [2]. In fact, the increased mortality risk among patients with type 2 diabetes in Western countries has mainly been attributed to CVD [2]. Nevertheless, the extent of increase in the mortality risk might vary among the populations of different countries [3]. In particular, this scenario might differ in Asian and other developing regions of the world, whereby infection and renal failure have been the main causes of death in people with diabetes [2]. In addition, evidence is also emerging that diabetes is associated with nonvascular conditions, especially its positive association with certain types of cancer [4, 5].

Although data on diabetes-related mortality has been available in other races and ethnic groups [1, 2, 6, 7], there is a lack of data on diabetes-related mortality that is representative of Asian population; this is particularly true of Korea, where diabetes remains a major health problem, with a reported prevalence of 11.0 % in 2013 [8]. A study using data from the Korea National Statistical Office revealed that mortality ascribed to diabetes has increased 3.5-fold between 1983 and 2001, which is a marked increase compared to that of other developed countries, such as Japan, the United States and the United Kingdom over a similar time period (1985–2000) [9]. However, the causes of death ascribed to diabetes-related mortality were diverse and some were even non-specific [9]. Hence, it is vital to obtain detailed and accurate information on the MRs and specific causes of death in the population of interest. Such knowledge could serve as a fundamental basis for establishing a national health plan, and could thereby minimize the diabetes-related hazards in public health in the long term.

Ideally, data should be randomly collected from unselected populations to avoid selection bias. However, most previous studies on mortality in diabetic populations including Korean studies have been confined to a particular setting, either in the community [10], or hospitals [2, 4, 11]. Although the data obtained from subjects in such limited settings can be easily recorded and handled, the findings are not representative of the national diabetic population. Recently, Korean National Health Insurance Service-National Sample Cohort (NHIS-NSC), a population-based cohort derived from nearly all Korean citizens, was established with an aim towards providing public health researchers and policy makers with representative, useful information regarding the utilization of health insurance and health examinations of the citizens [12].

In the present study, we aimed to obtain a reliable and comprehensive understanding of mortality in the Korean diabetic population by determining the MRs and causes of death from this national sample of Korea. Furthermore, we evaluated the excess mortality expressed as the standardized mortality ratio (SMR) in diabetic patients compared with that in the general population.

## Methods

### Study population

The NHIS was initiated in 1963 in Korea according to the National Health Insurance Act, and all Korean citizens were mandated to participate in this program [12]. Currently, the Korean NHIS maintains and manages all databases of Korea’s health service utilization. The detailed structure and function of NHIS is described elsewhere [12].

In the present study, we used data from the NHIS-NSC 2002–2013, which were released by the Korean NHIS in 2014. The data comprise a nationally representative random sample of 1,025,340 individuals, which accounts for approximately 2.2 % of the entire population in 2002 [12]. The data were built by using probabilistic sampling to represent an individual’s total annual medical expenses within each of 1476 strata defined by age, sex, eligibility status (employed or self-employed), and income level (20 quantiles for each eligibility status and medical-aid beneficiary) combinations via proportional allocation from the 46,605,433 Korean residents in 2002 [12, 13]. The NHIS-NSC is a semi-dynamically constructed cohort database; the cohort has been followed up to either the time of the participant’s disqualification from receiving health services due to death or emigration or until the end of the study period, whereas samples of newborn infants are included annually [12, 13]. The database contains eligibility and demographic information regarding health insurance as well as data on medical aid beneficiaries, medical bill details, medical treatment, disease histories and prescriptions; such data were constructed after converting insurance claim information to the first day of medical treatment.

From this cohort, we selected subjects recorded to have type 2 diabetes between 2002 and 2004. Type 2 diabetes was defined if anti-diabetic drugs were prescribed and the 10th revision of International Statistical Classification of Diseases, International Classification of Diseases (ICD)-10 codes E11 (non-insulin-dependent diabetes mellitus), E12 (malnutrition-related diabetes mellitus), E13 (other specified diabetes mellitus), or E14 (unspecified diabetes mellitus) was assigned as either principal or additional diagnosis. Antidiabetic drugs dispensed in the pharmacy during the study period in Korea consisted of six classes (i.e., sulfonylureas, biguanide, alpha-glucosidase inhibitor, thiazolidinediones, meglitinide and insulin) [14]. Incretin-based therapies (i.e. glucagon-like peptide -1 receptor agonists and dipeptidyl peptidase-4 inhibitors) were not introduced during the study period.

This diabetic cohort was followed up from the index date until the end of the study period (i.e., December 31, 2013), until the last year of qualification for those who were alive, or until the date of death for those who died. This study was approved by the NHIS inquiry commission. The personal privacy of each participant was protected by de-identification of the national insurance claims data for analysis. This study was also approved by the Institutional Review Board of the Asan Medical Center (IRB-No 2016-0149).

### Mortality data and causes of death

Information on mortality and cause of death were available for all subjects in the cohort; the latter was classified according to the Korean Standard Classification of Diseases and Causes of Death [15], based on the ICD-10, as provided by the Korean National Statistical Office. Diseases or conditions directly leading to death were adopted as the specific causes of death, and accordingly, they were classified as the following ICD-10 codes: malignant neoplasm (C00-97), diabetes mellitus (E11-14), diseases of the circulatory system (I00-99) and other causes (codes other than those mentioned above). Among the deaths due to diseases of the circulatory system (I00-99), deaths due to ischemic heart diseases (I20-25), and cerebrovascular diseases (I60-69) were further analyzed.

### Statistical analysis

We calculated the MR in each age group- and sex- by using the number of deaths as the numerator and the sum of person-years during the study period as the denominator. The MR ratios and their 95 % confidence intervals (CIs) for men versus women in each age stratum were estimated by Cox proportional hazard models.

Standardization was applied to calculate the SMRs, and the observed deaths were divided by the expected deaths for each age stratum and sex. Expected deaths were computed using the MR of the average of midyear population between 2002 and 2013, as reported by the Korean Statistical Information Service. (Statistics Korea. Causes of death statistics. 2002–2013) [16]. Byar’s approximation was used to calculate the 95 % CIs for the SMRs [17].

All statistical analyses were performed using SAS software (version 9.4, SAS Institute, Inc., Cary, NC, USA).

## Results

Of a total of 1,025,340 randomly selected individuals from the NHIS-NSC (comprising 2.2 % of the total eligible Korean population in 2002), we identified 29,807 diabetic individuals (15,625 men and 14,182 women) between 2002 and 2004 (Additional file 1: Table S1). Most of the enrolled subjects (70.3 %) were registered during the first year of this study. The NHIS database does not provide the exact age of each subject, but instead indicate the ages by 18 age groups (infants aged <1 year, ages 1–4 years, age groups with 5-year-intervals between 5 and 79 years, and ≥80 years) [12]. The majority of subjects (58.5 %) were in their 50s and 60s (Additional file 1: Table S1).

Over a total of 289,647.4 person-years of follow-up, 7103 subjects (3980 men and 3123 women, Additional file 1: Table S1) died and the crude MR was 26.7 and 22.2 per 1000 person-years in men and women, respectively (Table 1). The age- and sex-specific MR and SMRs, as well as the MR ratios for men versus women in each age stratum are listed in Table 1. The MR in the diabetic subjects tended to increase with age, from 5.7 per 1000 person-years in subjects aged 30–34 years to 153.6 per 1000 person-years in those aged ≥80 years in men; the corresponding value changed from 3.6 to 124.4 per 1000 person-years in women (Table 1). Furthermore, men with diabetes exhibited a higher risk of mortality (MR ratio: 1.21, 95 % CI 1.15–1.26; Table 1). The MR ratio for men versus women was highest in their early 40s (3.17, 95 % CI 1.98–5.07; Table 1). However, this sex-related difference in mortality risk was attenuated by increasing age after 50 years of age (Table 1).

**Table 1:** Age- and sex-specific mortality rates, standardized mortality ratios and mortality rate ratios for men versus women

Age (years)	Men					Women					MR ratio
	N	Deaths	Pearson-years	MR	SMR (95 % CI)	N	Deaths	Pearson-years	MR	SMR (95 % CI)	
30–34	369	22	3862.6	5.7	6.15 (3.86–9.32)	236	9	2523.8	3.6	6.65 (3.04–12.63)	1.60 (0.74–3.48)
35–39	801	51	8379.1	6.1	4.27 (3.18–5.61)	382	17	4050.7	4.2	6.01 (3.50–9.63)	1.44 (0.83–2.50)
40–44	1595	133	16,541.7	8.0	3.33 (2.79–3.95)	736	20	7849.5	2.5	2.60 (1.59–4.01)	3.17 (1.98–5.07)
45–49	2106	248	21,464.8	11.6	2.95 (2.59–3.34)	1097	63	11,628.9	5.4	3.82 (2.93–4.89)	2.13 (1.62–2.81)
50–54	2257	331	22,919.7	14.4	2.46 (2.20–2.74)	1509	102	16,119.3	6.3	3.13 (2.56–3.81)	2.29 (1.84–2.86)
55–59	2221	437	21,976.4	19.9	2.35 (2.13–2.58)	2040	206	21,615.1	9.5	3.26 (2.83–3.74)	2.10 (1.78–2.48)
60–64	2523	742	23,924.2	31.0	2.41 (2.24–2.59)	2615	430	27,033.4	15.9	3.38 (3.06–3.71)	1.98 (1.76–2.23)
65–69	1943	828	17,020.9	48.6	2.35 (2.19–2.52)	2362	593	23,313.7	25.4	3.06 (2.82–3.32)	1.96 (1.76–2.18)
70–74	984	556	7873.6	70.6	2.03 (1.87–2.21)	1721	702	15,490.2	45.3	2.83 (2.63–3.05)	1.61 (1.44–1.80)
75–79	534	386	3689.9	104.6	1.78 (1.61–1.97)	991	599	7697.1	77.8	2.43 (2.24–2.63)	1.38 (1.21–1.57)
80+	292	246	1601.9	153.6	1.23 (1.08–1.39)	493	382	3071.1	124.4	1.33 (1.20–1.47)	1.27 (1.08–1.49)
Overall	15,625	3980	149,254.6	26.7	2.20 (2.13–2.27)	14,182	3123	140,392.7	22.2	2.54 (2.45–2.63)	1.21 (1.15–1.26)

MR: mortality rate (1000 person-years), SMR: standardized mortality ratio, CI: confidence interval

Excess mortality among the diabetic subjects, relative to the general population, can be represented by SMRs. The overall SMR was 2.32 (Table 2), and the SMRs were found to be attenuated in the elderly subjects (Table 1). The SMR was greater in women than in men across most age groups (Table 1).

**Table 2:** Overall number (percentage) of deaths, mortality rates and standardized mortality ratios in specific causes deaths classified by ICD-10 codes

Causes of death	Men			Women			Total		
	Deaths (%)	MR	SMR (95 % CI)	Deaths (%)	MR	SMR (95 % CI)	Deaths (%)	MR	SMR (95 % CI)
All cause	3980 (100)	26.7	2.20 (2.13–2.27)	3123 (100)	22.2	2.54 (2.45–2.63)	7103 (100)	24.5	2.32 (2.27–2.38)
Malignant neoplasm	1177 (29.6)	7.9	1.83 (1.73–1.94)	582 (18.6)	4.1	1.70 (1.57–1.85)	1759 (24.8)	6.1	1.76 (1.67–1.84)
Diabetes mellitus	787 (19.8)	5.3	9.48 (8.83–10.17)	773 (24.8)	5.5	10.03 (9.34–10.77)	1560 (22.0)	5.4	9.73 (9.25–10.23)
Diseases of the circulatory system	753 (18.9)	5.0	2.04 (1.90–2.19)	811 (26.0)	5.8	2.35 (2.19–2.52)	1564 (22.0)	5.4	2.18 (2.07–2.29)
Ischemic heart disease	227 (5.7)	1.5	2.35 (2.05–2.68)	212 (6.8)	1.5	2.96 (2.58–3.39)	439 (6.2)	1.5	2.60 (2.37–2.86)
Cerebrovascular disease	378 (9.5)	2.5	1.91 (1.72–2.11)	421 (13.5)	3.0	2.21 (2.00–2.43)	799 (11.2)	2.8	2.04 (1.90–2.19)
All other causes	1263 (31.7)	8.5	1.76 (1.67–1.86)	957 (30.6)	6.8	2.06 (1.94–2.20)	2220 (31.3)	7.7	1.89 (1.81–1.97)

*MR* mortality rate (1000 person-years), *SMR* standardized mortality ratio, *CI* confidence interval

**Fig. 1:**
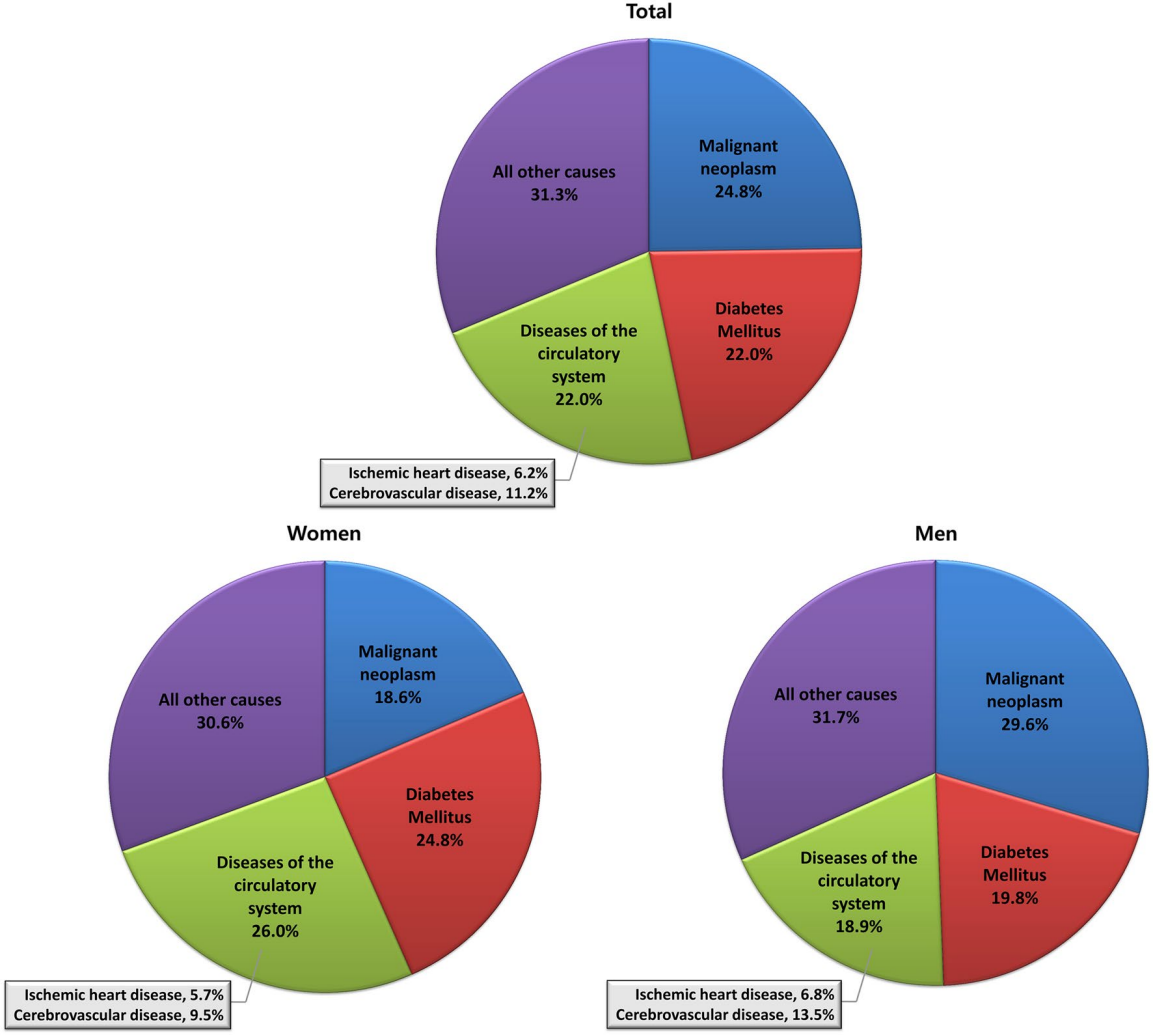
Overall mortality rates according to specific causes of death classified by ICD-10 codes

**Fig. 2:**
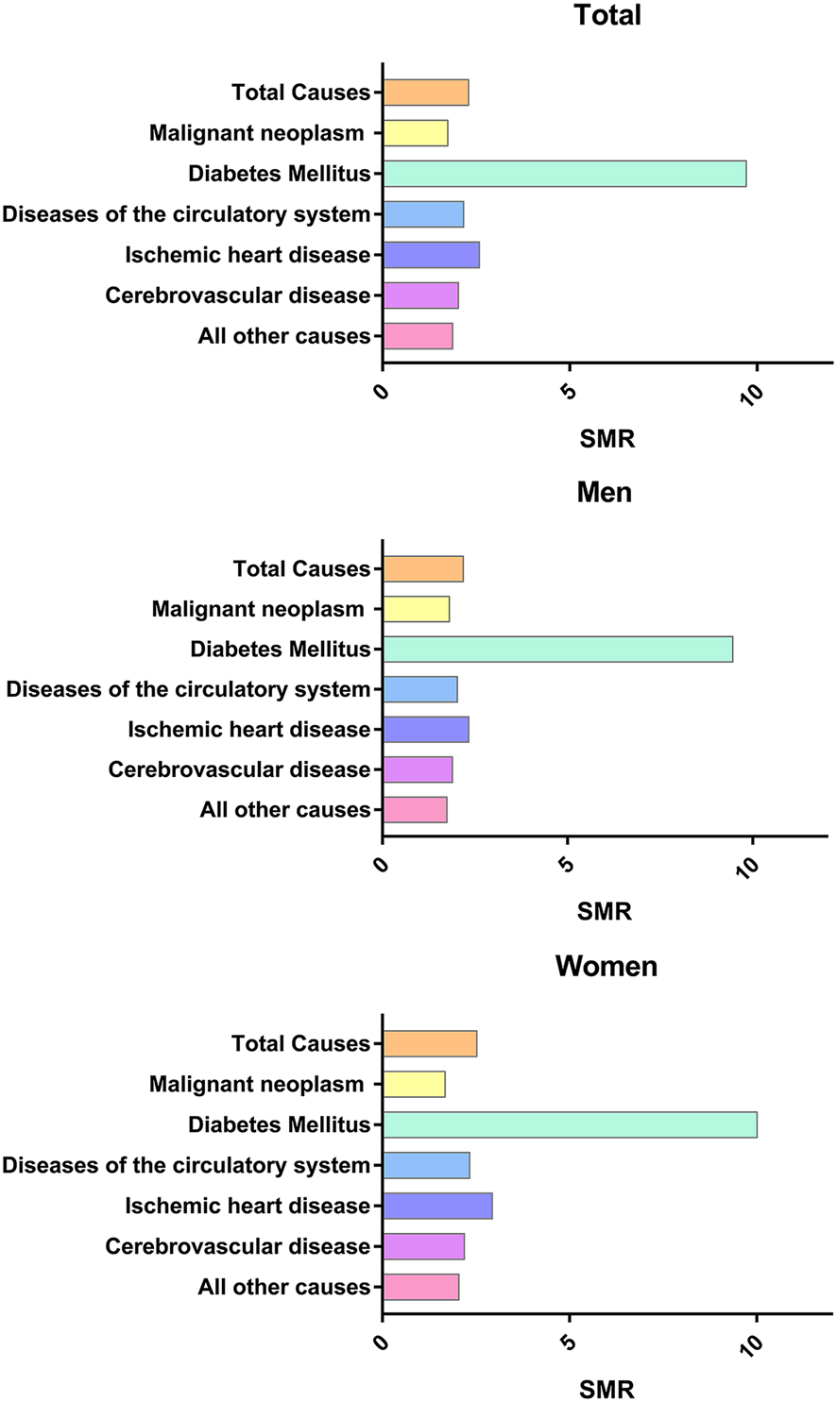
Standardized mortality ratios according to specific causes of death classified by ICD-10 codes

The observed numbers and percentages of the specific causes of death, as well as the SMRs for all causes and each specific cause of death according to sex are shown in Table 2, Figs. 1 and 2. Approximately 78.0 % of the diabetes-related deaths were not ascribed to diabetes in Korea. In fact, malignant neoplasm was the most common cause of death in this cohort (24.8 %, 6.1 per 1000 person-years) followed by the diseases of the circulatory system and diabetes. In particular, malignant neoplasm was the most common cause of death among men with diabetes (29.6 %, 7.9 per 1000 person-years, Table 2; Fig. 1). Diseases of the circulatory system were the most common cause of death among women with diabetes (26.0 %, 5.8 per 1000 person-years, Table 2; Fig. 1). In both sexes, the proportion of mortality cases ascribed to cerebrovascular disease was greater than that ascribed to ischemic heart disease (Table 2; Fig. 1). Furthermore, regardless of the causes of death listed in Table 2, diabetic subjects exhibited a significantly higher risk of mortality than the general population, as demonstrated by the SMRs (Table 2; Fig. 2). The excess mortality ascribed to ischemic heart disease as demonstrated by the SMRs was the highest of all causes, except for diabetes (2.60; 95 % CI 2.37–2.86; Table 2; Fig. 2).

## Discussion

This population-based cohort study provides a more accurate and less biased view of the issues regarding mortality in the Korean diabetic population. A comprehensive analysis of the MR, causes of death and SMR in subjects with type 2 diabetes, compared with those in the general population in Korea indicated that diabetic men had a higher mortality risk than diabetic women (Table 1). Moreover, diabetes was associated with excess mortality, which was primarily contributed by ischemic heart diseases, compared to that in the general population (Table 2; Fig. 2). Among the cases of mortality not ascribed to diabetes, malignant neoplasm was the most common cause of death (Table 2; Fig. 1).

Whereas previous studies investigating ethnic differences in mortality and major diabetic complications have enrolled Asian–Americans and Asian–Europeans with heterogeneous Asian backgrounds for comparison [18], our study enrolled Korean diabetic population residing in Korea, reflecting the genetic and cultural influences as well as the current features of Korean healthcare system. Nonetheless, despite the expected disparities, our results share several common findings reported from previous studies that enrolled both similar [7] and different [6, 19] ethnicities. The MRs according to age and sex observed in the present study (Table 1) were similar to those reported in previous population-based studies [6, 7]. For example, the MRs of diabetic patients in the US national cohort increased from 12.4 per 1000 person-years in those aged 25–44 years at baseline to 89.7 per 1000 person-years in those aged 65–74 years [6]. A study using the national sample of Taiwan also reported that the MR of diabetic patients increased from 14.1 per 1000 person-years in patients aged <45 years to 86.3 per 1000 person-years in those aged ≥75 years among diabetic men; and from 4.5 to 77.8 per 1000 person-years among diabetic women, respectively [7]. Similarly, the age-dependent increase in the MRs and the attenuation of the excess mortality (demonstrated by the SMRs) with increasing age (Table 1) were consistent with the findings of previous population-based studies [6, 7, 19]. In particular, Swedish National Diabetes Register reported much higher excess risk of death among patients younger than 55 years of age, as compared with controls in the same age group, despite a glycemic profile within the target range and normoalbuminuria [19]. Collectively, these results suggest that, regardless of ethnicity and the degree of glycemic control, special attention should be paid to the management of type 2 diabetes in a younger population to further improve their mortality outcomes.

With respect to sex-related differences, the MRs were relatively higher in diabetic men than in diabetic women (overall 1.21; Table 1), consistent with the findings from other studies [6, 7, 20]. In contrast, the overall SMR was greater in women (2.54 [95 % CI, 2.45–2.63]) than in men (2.20 [2.13–2.27]; Table 1) in our study, which suggests that Korean women are more likely to be vulnerable to the malicious effects of diabetes as compared to Korean diabetic men, once they are diagnosed with it.

In our current study, we found that only 22 % of the subjects had diabetes mellitus coded as the primary cause of death on their death certificates (Table 2; Fig. 1). Although the direct cause of such diabetes-related death remains unclear, the findings of a national cohort study of Taiwan has provided an important clue [7]. In this study, the authors postulated that individuals with diabetes as the recorded cause of death are more likely to have died due to CVD than due to cancer, since it is much more plausible for clinicians to detect and code for cancer on the death certificate if the patient had died from cancer despite the co-existence of diabetes or its related complications [7]. Moreover, if we consider that the patients in the present study actually died due to CVD rather than diabetes, as reported, then the proportion of such subjects would sum up to 44 %, which is similar to the values from other nationwide cohort studies [6, 7, 21]. Nevertheless, the limitations of mortality record-based studies should not be overlooked, and this issue requires further investigation.

Besides diabetes, malignant neoplasm (24.8 %) and diseases of the circulatory system (22.0 %) including ischemic heart diseases and cerebrovascular diseases were the two most common causes of death in both sexes (Table 2; Fig. 1). With regard to sex-related differences, we found that the most common cause of death was malignant neoplasm among diabetic men and diseases of the circulatory system among diabetic women (Table 2; Fig. 1). Similar trends were noted in a Taiwanese study [7], wherein the sum of percentages of deaths attributed to a composite of cardiopulmonary diseases, stroke, and disease of arteries, arterioles, and capillaries was greater than the percentages of deaths due to cancer in diabetic women, whereas the converse was true for diabetic men [7].

Epidemiologic evidence suggests that individuals with diabetes are at a significantly higher risk for many forms of cancer [5]. Moreover, findings from other observational studies indicate that some medications used to treat hyperglycemia might also be associated with either an increased or reduced risk of cancer [22, 23]. The relative risks due to diabetes were greater (approximately twofold or higher) for malignant neoplasm of the liver, pancreas, and endometrium, and lower (approximately 1.2- to 1.5-fold higher) for malignant neoplasm of the colon and rectum, breast, and bladder [23]. Among the deaths ascribed to malignant neoplasm in the present study, malignant neoplasm of the liver and intrahepatic bile ducts was the most common cause of death (5.1 %, 1.2 per 1000 person-years; Supplementary Table S2). Despite the high prevalence of hepatitis B or C in Korea [24, 25], this finding further support that diabetes-related factors including steatosis, nonalcoholic fatty liver disease, and cirrhosis might also enhance the susceptibility to liver cancer [26]. With regard to SMRs, death due to malignant neoplasm of the pancreas showed the highest SMR, compared to that in the general population (2.59, 95 % CI 2.19–3.05. Additional file 1: Table S2). The high MRs due to malignancy seen in our present study support the previous epidemiologic evidence regarding the increased risk of malignancy in people with diabetes [5]; this theory is also supported by the marked increases in cancer-related death (from 4.7 to 21.9 %) in Korean individuals with type 2 diabetes over the past 10 years [4].

As supported by numerous epidemiologic and prospective studies, type 2 diabetes significantly increases the absolute risk of developing CVDs in both Western [27] and Asian [28] populations. In Western countries, CVD is the leading cause of death among individuals with diabetes, and the risk of atherosclerotic diseases is increased by two to threefold [2]. Likewise, the proportion of deaths attributed to CVD among individuals with diabetes from the Asia-Pacific region was similar to that observed in other countries [2]. In particular, large-scale nationwide studies and multinational analyses have indicated that, heart disease is a more common cause of death than stroke in most Western countries [7], whereas stroke is a more common cause of death in Asian countries [2]. In the present study, the MRs ascribed to cerebrovascular diseases were greater than those ascribed to ischemic heart disease in both genders (Table 2; Fig. 1). This distinctive finding can be explained by the fact that, in Korea, stroke remains a major health burden and that the risk factors for stroke are highly prevalent [29]. For the period between 2001 and 2009, the rate of admission for stroke, including hemorrhagic stroke, as the primary diagnosis remained stable, whereas the admission rates for ischemic stroke substantially increased in Korea [29]. Moreover, based on a prospective cohort study performed in the 1990s and early 2000s, hypertension had the highest population attributable risk (PAR) for stroke, which was even greater than that for ischemic heart disease. On the contrary, diabetes had a relatively low PAR for stroke in the Korean population [29]. These findings suggest that this high prevalence of stroke and the relatively low level of contribution of diabetes to the risk of stroke in the Korean population might have led to the higher MR ascribed to cerebrovascular diseases than those ascribed to ischemic heart diseases (Table 2; Fig. 1).

A comparison of SMRs for the common cause of death in individuals with diabetes showed that diseases of the circulatory system including ischemic heart disease and cerebrovascular disease were a major contributor of excess mortality in the diabetic population, compared to that of the general population (Table 2; Fig. 2). In particular, Fig. 2 illustrates a greater excess mortality ascribed to CVD in women than in men with diabetes. Although the reason underlying this sex-related difference is unclear, a relative loss of protection from cardiovascular deaths among women with diabetes might be a possible cause, as a similar loss of cardiovascular advantage has been previously observed in data from the US and the United Kingdom [30]. In addition, among the causes other than malignant neoplasm, diabetes and diseases of the circulatory system, renal failure showed the greatest association with the excess MR (3.0 % with an SMR of 4.44 [95 % CI 3.84–5.10]; Additional file 1: Table S3). Because reduced estimated glomerular filtration rate is a strong and independent risk factor for death and CVD [31], this finding indicates that Koreans with diabetes have a higher risk of dying from severe renal disease or its related conditions, than the general population.

Multiple factors such as age, sex, body mass index, unhealthy metabolic profile including triglycerides levels and impaired glucose tolerance state, and the existence of comorbidities are associated with mortality in patients with type 2 diabetes [32–36]. Among these, the association between treatment-related factors and mortality in diabetic patients has been recently reported [37]. For example, the implementation of a multidisciplinary personalized intervention program was associated with lower CVD and all-cause mortality over 3-year follow-up in diabetic patients in Hong Kong [37]. On the other hand, the use of chronic medications to treat diabetes and comorbidities was associated with higher micro-/macrovascular event, hospitalization, and death risk [38]. Moreover, a recent meta-analysis revealed that diabetes mellitus, especially with the requirement of insulin treatment was associated with significantly higher short and long-term adverse cardiovascular outcomes after percutaneous coronary intervention compared to those who were not on insulin therapy [39, 40]. Therefore, we performed additional analyses to determine whether the use of insulin in our cohort led to worse mortality-related outcomes. Consequently, insulin users showed much higher MRs and SMRs across all age groups, in both sexes (Additional file 1: Tables S4, S5). Direct complications from insulin administration such as weight gain or hypoglycemia might have contributed to higher mortality in this subpopulation; however, given that insulin therapy is generally administered 10–15 years after diagnosis [41], it is more plausible that the requirement of insulin therapy reflects the longer and severe duration of diabetes [39]. Nevertheless, in line with previous findings, our results suggest that insulin treatment is associated with higher mortality in type 2 diabetic patients, and thus, prognosis should be assessed differently when managing diabetic patients on insulin therapy.

The major strengths of our present study are the utilization of a large and national sample of diabetic patients and the use of national registry data that allowed for a comprehensive understanding and ascertainment of information on mortality. However, our analyses had several limitations of note. First, our study only included people with type 2 diabetes, and hence did not reflect information on mortality in people with type 1 diabetes in Korea. However, a much greater proportion of the diabetic population is diagnosed with type 2 diabetes in Korea, and thus, our results reflect the majority of Korean diabetic population [42, 43]. Second, as we defined type 2 diabetes based on the prescription of anti-diabetic drugs as well as the presence of ICD-10 codes, type 2 diabetic patients being controlled with lifestyle (i.e. diet and exercise) modification and those who were undiagnosed prior to the analysis might have been misclassified as not having diabetes. Therefore, it was unable to analyze the cause of death in this subpopulation. Third, the potentially underreported cases of death ascribed to diabetes and the misclassification of some causes of death might have interfered with a more precise analysis. Our findings showed that more than three quarters of the diabetes-related deaths would not be ascribed to diabetes in Korea (Table 2; Fig. 1). However, any death certificate-based mortality study [21] has certain limitations due to the difficulty in authenticating the cause of death [30, 44–46]; nevertheless, we attempted to overcome this limitation by assessing a large-scale, nationally representative dataset. Lastly, we could not predict the severity of diabetes by serologic markers such as HbA1c because HbA1c screening is not included in the national health screening program in Korea. However, we additionally ran subgroup analysis of patients with and without the requirement of insulin therapy, to identify mortality-related outcomes according to the severity of diabetes.

In conclusion, more than 75 % of the diabetes-related deaths in Korea were in fact recorded not to be ascribed to diabetes. Moreover, diabetic men have a higher mortality risk than diabetic women, and diabetic patients have excess mortality compared to the general population. Besides diabetes, malignant neoplasm was most common among all causes of death, and ischemic heart disease was primarily responsible for excess mortality in individuals with diabetes compared to the general Korean population. These findings highlight the potential benefits of controlling cardiovascular risk factors and cancer screening in the diabetic population in Korea. However, the fact that 31.3 % of death was attributed to other causes should also not be ignored. Further long-term investigation of various outcomes in this population may help confirm such beneficial effects.

## Additional file

10.1186/s12933-016-0451-0 Additional tables.

## Abbreviations

CI: confidence interval
CVD: cardiovascular disease
ICD: International Classification of Diseases
MR: mortality rate
NHIS-NSC: National Health Insurance Service-National Sample Cohort
SMR: standardized mortality rate
