# Supplementary material for: Mortality and causes of death in a national sample of type 2 diabetic patients in Korea from 2002 to 2013
Source: Cardiovasc Diabetol. 2016 Sep 13;15(1):131. doi: 10.1186/s12933-016-0451-0 (PMC5020435; doi:10.1186/s12933-016-0451-0)
Supplement: Supplementary file 1 — 10.1186/s12933-016-0451-0 Additional tables. [file 12933_2016_451_MOESM1_ESM.docx]

**Supplementary Table S1.**Characteristics of patients with type 2 diabetes from the NHIS-NSC database (2002-2004)

| **Characteristics** | **Total** | **Men** | **Women** |
| --- | --- | --- | --- |
|  | **(N=29,807)** | **(N=15,625)** | **(N=14,182)** |
| **Year (N, %)** |  |  |  |
| 2002 | 20,996 (70.3) | 10,528 (67.4) | 10,438 (73.6) |
| 2003 | 5,224 (17.5) | 2,961 (19.0) | 2,263 (16.0) |
| 2004 | 3,617 (12.1) | 2,136 (13.7) | 1,481 (10.4) |
| **Age group (N, %)** |  |  |  |
| 30–34 | 605 (2.0) | 369 (2.4) | 236 (1.7) |
| 35–39 | 1183 (4.0) | 801 (5.1) | 382 (2.7) |
| 40–44 | 2331 (7.8) | 1,595 (10.2) | 736 (5.2) |
| 45–49 | 3203 (10.7) | 2,106 (13.5) | 1,097 (7.7) |
| 50–54 | 3766 (12.6) | 2,257 (14.4) | 1,509 (10.6) |
| 55–59 | 4261 (14.3) | 2,221 (14.2) | 2,040 (14.4) |
| 60–64 | 5138 (17.2) | 2,523 (16.1) | 2,615 (18.4) |
| 65–69 | 4305 (14.4) | 1,943 (12.4) | 2,362 (16.7) |
| 70–74 | 2705 (9.1) | 984 (6.3) | 1,721 (12.1) |
| 75–79 | 1525 (5.1) | 534 (3.4) | 991 (7.0) |
| 80+ | 785 (2.6) | 292 (1.9) | 493 (3.5) |
| **Survival** |  |  |  |
| Alive | 22,704 (76.2) | 11,645 (74.5) | 11,059 (78.0) |
| Dead | 7,103 (23.8) | 3,980 (25.5) | 3,123 (22.0) |
| **Follow-up duration**  (years, mean ± SD) |  |  |  |
| Total subjects | 9.72 ±3.01 | 9.55 ± 3.10 | 9.90 ± 2.91 |
| Alive | 10.92 ±1.63 | 10.88 ± 1.59 | 10.97 ± 1.66 |
| Dead | 5.87 ±3.20 | 5.68 ± 3.17 | 6.12 ±3.22 |

**Supplementary Table S2.**Overall number (percentage) of deaths, mortality rates and standardized mortality ratios according to cancer types,

classified by the ICD-10 codes

|  | **Men** |  |  | **Women** |  |  | **Total** |  |  |
| --- | --- | --- | --- | --- | --- | --- | --- | --- | --- |
| **Causes of death** | **Deaths (%)** | **MR** | **SMR (95% CI)** | **Deaths (%)** | **MR** | **SMR (95% CI)** | **Deaths (%)** | **MR** | **SMR (95% CI)** |
| **All cause** | 3,980 (100) | 26.7 | 2.20 (2.13-2.27) | 3,123 (100) | 22.2 | 2.54 (2.45-2.63) | 7,103 (100) | 24.5 | 2.32 (2.27-2.38) |
| **Malignant neoplasm (Total)** | 1,177 (29.6) | 7.9 | 1.83 (1.73-1.94) | 582 (18.6) | 4.1 | 1.70 (1.57-1.85) | 1,759 (24.8) | 6.1 | 1.76 (1.67-1.84) |
| **Malignant neoplasm of the stomach (C16)** | 157 (3.9) | 1.1 | 1.52 (1.29-1.78) | 70 (2.2) | 0.5 | 1.51 (1.18 – 1.91) | 227 (3.2) | 0.8 | 1.49 (1.30-1.69) |
| **Malignant neoplasm of the colon (C18)** | 55 (1.4) | 0.4 | 1.87 (1.41-2.43) | 49 (1.6) | 0.3 | 2.01 (1.49-2.66) | 104 (1.5) | 0.4 | 1.91 (1.56-2.31) |
| **Malignant neoplasm of the colon and rectum, rectal ampulla, and anus and anal canal (C19**–**21)** | 41 (1.0) | 0.3 | 1.64 (1.18-2.23) | 30 (1.0) | 0.2 | 1.93 (1.30-2.76) | 71 (10) | 0.2 | 1.73 (1.36-2.18) |
| **Malignant neoplasm of the liver and intrahepatic bile ducts (C22)** | 271 (6.8) | 1.8 | 2.11 (1.86-2.37) | 88 (2.8) | 0.6 | 2.06 (1.65-2.53) | 359 (5.1) | 1.2 | 2.10 (1.89-2.33) |
| **Malignant neoplasm of the pancreas (C25)** | 89 (2.2) | 0.6 | 2.79 (2.24-3.43) | 60 (1.9) | 0.4 | 2.39 (1.83-3.08) | 149 (2.1) | 0.5 | 2.59 (2.19-3.05) |
| **Malignant neoplasm of the bronchus and lung (C34)** | 271 (6.8) | 1.8 | 1.66 (1.47-1.87) | 77 (2.5) | 0.5 | 1.42 (1.12-1.78) | 348 (4.9) | 1.2 | 1.54 (1.39-1.71) |

MR: mortality rate(1,000 person-years), SMR: standardized mortality ratio, CI: confidence interval

**Supplementary Table S3.**Overall number (percentage) of deaths, mortality rates and standardized mortality ratios other than diabetes mellitus, malignant neoplasms, and diseases of the circulatory system

|  | **Men** |  |  | **Women** |  |  | **Total** |  |  |
| --- | --- | --- | --- | --- | --- | --- | --- | --- | --- |
| **Causes of death** | **Deaths (%)** | **MR** | **SMR (95% CI)** | **Deaths (%)** | **MR** | **SMR (95% CI)** | **Deaths (%)** | **MR** | **SMR (95% CI)** |
| **Total other causes** | 1,263 (31.7) | 6.7 | 1.79 (1.68-1.91) | 957 (30.6) | 6.8 | 2.06 (1.94-2.20) | 2,220 (31.3) | 7.7 | 1.89 (1.81-1.97) |
| **Disease of the digestive system (K00-92)** | 214 (5.4) | 1.4 | 1.90 (1.66-2.18) | 100 (3.2) | 0.7 | 2.51 (2.04-3.06) | 314 (4.4) | 1.1 | 2.11 (1.88-2.36) |
| **Diseases of liver (K70-76)** | 176 (4.4) | 1.2 | 1.94 (1.67-2.25) | 43 (1.4) | 0.3 | 2.05 (1.49-2.77) | 219 (3.1) | 0.8 | 2.03 (1.77-2.32) |
| **Disease of the respiratory system (A00-B99)** | 186 (4.7) | 1.2 | 1.60 (1.38-1.84) | 134 (4.3) | 1.0 | 4.71 (4.21-5.25) | 320 (4.5) | 1.1 | 1.70 (1.52-1.90) |
| **Pneumonia (J12-18)** | 65 (1.6) | 0.4 | 2.07 (1.60-2.64) | 58 (1.9) | 0.4 | 5.32 (4.42-6.35) | 123 (1.7) | 0.4 | 2.23 (1.86-2.66) |
| **Chronic lower respiratory diseases (J40-47)** | 81 (2.0) | 0.5 | 1.36 (1.08-1.70) | 47 (1.5) | 0.3 | 1.49 (1.10-1.99) | 128 (1.7) | 0.4 | 1.38 (1.15-1.64) |
| **Diseases of the genitourinary system (N00-98)** | 125 (3.1) | 0.8 | 4.77 (3.97-5.68) | 97 (3.1) | 0.7 | 3.75 (3.04-4.58) | 222 (3.1) | 0.8 | 4.24 (3.70-4.84) |
| **Renal failure (N17-19)** | 118 (3.0) | 0.8 | 5.09 (4.21-6.09) | 80 (2.6) | 0.6 | 3.79 (3.01-4.72) | 198 (2.8) | 0.7 | 4.44 (3.84-5.10) |
| **Infectious diseases (A00-B99)** | 83 (2.1) | 0.6 | 1.84 (1.47-2.28) | 54 (1.7) | 0.4 | 2.02 (1.52-2.64) | 137 (1.9) | 0.5 | 1.92 (1.67-2.27) |
| **Diseases of the nervous system (G00-98)** | 62 (1.6) | 0.4 | 2.28 (1.75-2.92) | 52 (1.7) | 0.4 | 1.71 (1.28-2.24) | 114 (1.6) | 0.4 | 1.97 (1.63-2.37) |
| **Mental and behavioral disorders (F0-99)** | 22 (0.6) | 0.1 | 0.90 (0.56-1.36) | 32 (1.0) | 0.2 | 1.33 (0.91-1.88) | 54 (0.8) | 0.2 | 1.11 (0.84-1.45) |
| **Diseases of musculo-skeletal system and connective tissue (M00-99)** | 12 (0.3) | 0.1 | 1.82 (0.94-3.18) | 16 (0.5) | 0.1 | 1.16 (0.66-1.88) | 28 (0.4) | 0.1 | 1.37 (0.91-1.97) |
| **Other endocrine, nutritional and metabolic diseases (excluding E11-14, E00-90)** | 7 (0.2) | 0.0 | 1.56 (0.62-3.21_ | 9 (0.3) | 0.1 | 2.14 (0.98-4.07) | 16 (0.2) | 0.1 | 1.85 (1.06-3.00) |
| **Hematologic or Immune disorders (D50-89)** | 6 (0.2) | 0.0 | 2.31 (0.84-5.03) | 3 (0.1) | 0.0 | 1.04 (0.21-3.03) | 9 (0.1) | 0.0 | 1.63 (0.74-3.09) |
| **Diseases of the skin and subcutaneous tissue (L00-98)** | 2 (0.1) | 0.0 | 1.19 (0.13-4.28) | 4 (0.1) | 0.0 | 1.48 (0.40-3.79) | 6 (0.1) | 0.0 | 1.36 (0.50-2.96) |
| **Congenital malformations, deformations and chromosomal abnormalities (Q00-99)** | - | 0.0 | 0.0 | 2 (0.1) | 0.0 | 2.94 (0.33-10.61) | 2 (0.0) | 0.0 | 1.61 (0.18-5.80) |
| **Symptoms, signs, and abnormal clinical and laboratory findings (R00-99)** | 163 (4.1) | 1.1 | 1.45 (1.23-1.69) | 214 (6.9) | 1.5 | 1.72 (1.49-1.96) | 377 (5.3) | 1.3 | 1.58 (1.42-1.75) |
| **All other causes (injury, poisoning and certain other consequences of external causes, S00-T98)** | 376 (9.4) | 2.5 | 1.64 (1.47-1.81) | 228 (7.3) | 1.6 | 2.42 (2.12-2.75) | 604 (8.5) | 2.1 | 1.89 (1.74-2.04) |

MR: mortality rate(1,000 person-years), SMR: standardized mortality ratio, CI: confidence interval

**Supplementary Table S4.** Age- and sex-specific mortality rates, standardized mortality ratios and mortality rate ratios for men versus women (insulin users only)

|  | **Men** |  |  |  |  | **Women** |  |  |  |  |  |
| --- | --- | --- | --- | --- | --- | --- | --- | --- | --- | --- | --- |
| **Age**  **(years)** | **N** | **Deaths** | **Pearson-years** | **MR** | **SMR (95% CI)** | **N** | **Deaths** | **Pearson-years** | **MR** | **SMR (95% CI)** | **MR ratio** |
| 30-34 | 55 | 5 | 571.6 | 8.7 | 9.45 (3.05-22.06) | 75 | 5 | 807.3 | 6.2 | 11.55 (3.72-26.96) | 1.43 (0.41-4.95) |
| 35-39 | 101 | 14 | 1025.6 | 13.7 | 9.57 (5.23-16.07) | 61 | 10 | 632.9 | 15.8 | 22.64 (10.84-41.65) | 0.86 (0.38-1.94) |
| 40-44 | 186 | 38 | 1823.0 | 20.8 | 8.63 (6.11-11.85) | 114 | 10 | 1193.8 | 8.4 | 8.54 (4.09-15.71) | 2.48 (1.24-4.98) |
| 45-49 | 246 | 65 | 2293.2 | 28.3 | 7.24 (5.59-9.23) | 150 | 18 | 1562.6 | 11.5 | 8.12 (4.81-12.83) | 2.43 (1.44-4.09) |
| 50-54 | 289 | 108 | 2495.2 | 43.3 | 7.36 (6.04-8.89 | 210 | 39 | 2175.0 | 17.9 | 8.88 (6.32-12.14) | 2.42 (1.68-3.50) |
| 55-59 | 343 | 130 | 3083.7 | 42.2 | 4.97 (5.16-5.91) | 311 | 72 | 3164.1 | 22.8 | 7.79 (6.09-9.81) | 1.88 (1.41-2.50) |
| 60-64 | 401 | 196 | 3291.4 | 59.5 | 4.63 (4.01-5.33) | 422 | 142 | 3998.5 | 35.5 | 7.54 (6.35-8.88) | 1.70 (1.37-2.11) |
| 65-69 | 330 | 207 | 2403.8 | 86.1 | 4.16 (3.61-4.77) | 418 | 179 | 3628.2 | 49.3 | 5.93 (5.10-6.87) | 1.77 (1.45-2.16) |
| 70-74 | 198 | 142 | 1372.0 | 103.5 | 2.98 (2.51-3.51) | 296 | 169 | 2346.7 | 72.0 | 4.50 (3.85-5.23) | 1.48 (1.18-1.85) |
| 75-79 | 102 | 84 | 550.2 | 152.7 | 2.60 (2.07-3.22) | 159 | 118 | 1002.6 | 117.7 | 3.67 (3.04-4.40) | 1.30 (0.99-1.73) |
| 80+ | 57 | 51 | 228.6 | 223.1 | 1.79 (1.33-2.35) | 84 | 79 | 346.7 | 227.8 | 2.43 (1.92-3.03) | 1.03 (0.72-1.48) |
| Overall | 2,308 | 1,040 | 19138.2 | 54.3 | 4.05 (3.81-4.30) | 2,300 | 841 | 20858.4 | 40.3 | 4.97 (4.64-5.32) | 1.35 (1.24-1.48) |

MR: mortality rate (1,000 person-years), SMR: standardized mortality ratio, CI: confidence interval

**Supplementary Table S5.** Age- and sex-specific mortality rates, standardized mortality ratios and mortality rate ratios for men versus women (insulin non-users)

|  | **Men** |  |  |  |  | **Women** |  |  |  |  |  |
| --- | --- | --- | --- | --- | --- | --- | --- | --- | --- | --- | --- |
| **Age**  **(years)** | **N** | **Deaths** | **Pearson-years** | **MR** | **SMR (95% CI)** | **N** | **Deaths** | **Pearson-years** | **MR** | **SMR (95% CI)** | **MR ratio** |
| 30-34 | 314 | 17 | 3291.0 | 5.2 | 5.58 (3.25-8.94) | 161 | 4 | 1716.5 | 2.3 | 4.35 (1.17-11.13) | 2.22 (0.75-6.59) |
| 35-39 | 700 | 37 | 7353.6 | 5.0 | 3.53 (2.48-4.86) | 321 | 7 | 3417.8 | 2.0 | 2.94 (1.18-6.05) | 2.44 (1.09-5.48) |
| 40-44 | 1,409 | 95 | 14718.7 | 6.5 | 2.67 (2.16-3.27) | 622 | 10 | 6655.7 | 1.5 | 1.53 (0.73-2.82) | 4.31 (2.25-8.28) |
| 45-49 | 1,860 | 183 | 19171.7 | 9.5 | 2.44 (2.10-2.82) | 947 | 45 | 10066.3 | 4.5 | 3.15 (2.30-4.22) | 2.14 (1.55-2.97) |
| 50-54 | 1,968 | 223 | 20424.4 | 10.9 | 1.86 (1.62-2.12) | 1,299 | 63 | 13944.3 | 4.5 | 2.24 (1.72-2.86) | 2.43 (1.84-3.21) |
| 55-59 | 1,878 | 307 | 18892.7 | 16.2 | 1.92 (1.71-2.14) | 1,729 | 134 | 18451.0 | 7.3 | 2.49 (2.08-2.94) | 2.26 (1.84-2.76) |
| 60-64 | 2,122 | 546 | 20632.8 | 26.5 | 2.06 (1.89-2.24) | 2,193 | 288 | 23034.9 | 12.5 | 2.65 (2.36-2.98) | 2.15 (1.86-2.48) |
| 65-69 | 1,613 | 621 | 14617.1 | 42.5 | 2.05 (1.89-2.22) | 1,944 | 414 | 19685.5 | 21.0 | 2.53 (2.29-2.79) | 2.08 (1.84-2.36) |
| 70-74 | 786 | 414 | 6501.7 | 63.7 | 1.83 (1.66-2.02) | 1,425 | 533 | 13143.6 | 40.6 | 2.53 (2.32-2.76) | 1.62 (1.43-1.85) |
| 75-79 | 432 | 302 | 3139.7 | 96.2 | 1.64 (1.46-1.83) | 832 | 481 | 6694.5 | 71.9 | 2.24 (2.05-2.45) | 1.38 (1.19-1.59) |
| 80+ | 235 | 195 | 1373.3 | 142.0 | 1.14 (0.98-1.31) | 409 | 303 | 2724.3 | 111.2 | 1.19 (1.06-1.33) | 1.32 (1.10-1.58) |
| Overall | 13,317 | 2,940 | 130116.5 | 22.6 | 1.89 (1.82-1.96) | 11,882 | 2,282 | 119534.3 | 19.1 | 2.16 (2.07-2.25) | 1.19 (1.13-1.26) |

MR: mortality rate (1,000 person-years), SMR: standardized mortality ratio, CI: confidence interval
